# CD226 implicated in Akt-dependent apoptosis of CD4^+^ T cell contributes to asthmatic pathogenesis

**DOI:** 10.1038/s41419-024-07080-z

**Published:** 2024-09-30

**Authors:** Yuan Zhang, Yang Xie, Xuexin Zhang, Chujun Duan, Jingchang Ma, Yuling Wang, Yilin Wu, Niqi Shan, Kun Cheng, Ran Zhuang, Ka Bian

**Affiliations:** 1 Department of Otolaryngology Head and Neck Surgery, Tangdu Hospital, Fourth Military Medical University, Xi’an, Shaanxi China; 2 Department of Immunology, Fourth Military Medical University, Xi’an, Shaanxi China; 3 Institute of Medical Research, Northwestern Polytechnical University, Xi’an, Shaanxi China

**Keywords:** Immunological disorders, Experimental models of disease

## Abstract

Asthma is a chronic airway inflammatory disease in which CD4^+^ T cell dysregulation occurs. Here, we investigated the molecular role and clinical significance of CD226, a costimulatory molecule of T lymphocytes, in the development of allergic asthma. Our results revealed that the expression of CD226 was significantly increased in CD4^+^ effector T cells, especially in T helper (Th) 2 cells and Th17 cells in patients with asthma. Moreover, CD4^+^ T cell-specific *Cd226*-knockout mice were generated and together with littermates were challenged with ovalbumin (OVA) to establish a model of allergic asthma. We found that CD226 deficiency in CD4^+^ T cells mitigated lung inflammation, IgE production, and eosinophil infiltration and reduced airway remodeling in experimental allergic asthma. However, the impact of CD226 on asthma was independent of Treg cell modulation. Through RNA-seq data analysis, the apoptosis pathway was screened. Mechanistically, CD226 deletion promoted CD4^+^ T cell late apoptosis via the activation of Caspase-3 in an Akt-dependent manner. Furthermore, blocking CD226 signaling with a recombinant fusion protein attenuated asthma features in mice and achieved a good therapeutic effect. Overall, this study revealed a unique role of CD226 in CD4^+^ T cell regulation in asthma pathogenesis. Therefore, targeting CD226 may provide new insights into the clinical treatment of asthma.

## Introduction

Asthma is a common disease and a major public health concern worldwide that is associated with excess morbidity, mortality, and economic costs [1]. The prevalence of asthma has increased substantially in recent years, and >400 million individuals may be affected by asthma by 2025 [2, 3]. Chronic allergic asthma accounts for most cases of asthma and is characterized by T helper (Th) 2-type inflammation, enabling the infiltration of eosinophils, followed by airway hyperresponsiveness and tissue remodeling [4, 5]. Asthma inflammation is characterized by allergen-specific IgE antibodies and may be followed by atopy [6]. Although immune responses are recognized to play a crucial role in the occurrence and development of asthma, the complex mechanisms underlying immune regulation remain incompletely elucidated, necessitating further investigation to guide immunotherapy.

T cell function is regulated by various immune signals, including cytokines and costimulatory signals. CD226 (also known as DNAM-1 and PTA-1) is a costimulatory receptor expressed on most T cells, natural killer (NK) cells, and monocytes [7, 8]. CD226 competes with T-cell immunoreceptor with Ig and immunoreceptor tyrosine-based inhibitory domains (TIGIT) on immune cells for binding to CD155/CD112; and upon ligation with the ligands, CD226 and TIGIT show reciprocal functions [9]. CD226 and TIGIT are expressed at varying levels depending on the T cell subsets and activation states [10], and imbalance of the CD226/TIGIT signal is involved in the pathogenesis of cancer, autoimmune diseases, and inflammatory responses [9, 11]. CD226 phosphorylation at Y319 and Y322 is needed for the downstream signaling activation, including that of Erk, p38, and Akt, and for corresponding NK and T cell responses [12, 13].

CD226 has been demonstrated as an anti-apoptotic molecule that may be involved in the development of murine thymocytes and the activation of regulatory T (Treg) cells [14–18]. Despite the critical roles of this checkpoint receptor in T cell immunoregulation, the effects of CD226 against allergic diseases, such as asthma, and the underlying mechanisms remain largely unknown. The potential mechanisms of CD226 have been recently revealed in alleviating airway hyperresponsiveness via regulating type 2 innate lymphoid cell (ILC2) function [19], indicating the potential role of CD226 in allergic airway responses. Herein, we first evaluated the expression levels of CD226 on CD4^+^ T cells in patients with asthma. Next, we used a conditional knockout (CKO) strategy and observed that the loss of CD226 in CD4^+^ T cells alleviated ovalbumin (OVA)-induced allergic asthma in mice, and CD226 played a critical role in regulating CD4^+^ T cell apoptosis. In addition, we demonstrated that the CD226-Fc recombinant protein exhibited a protective effect against allergic asthma in mice. In the present study, our data for the first time demonstrated that CD226, as a pivotal costimulatory molecule present on T cells, can potentially serve as a novel therapeutic target for allergic asthma.

## Materials and methods

### Human samples

To detect significant differences in CD226 expression between asthma and healthy groups, we set α at 0.05 and β at 0.80. Based on the pre-experimental results, we estimated that the mean proportions of CD226^+^ CD4^+^ T cells in the two groups were 85.0% and 80.5% respectively, with a standard deviation of 4.2057. At least 15 participants were required for each group according to the sample size calculation formula $n=2\frac{{({Z}_{\frac{\alpha }{2}}+{Z}_{\beta })}^{2}{\sigma }^{2}}{{({\mu }_{1}-{\mu }_{2})}^{2}}$. Patients with asthma and healthy controls were recruited at the Tangdu Hospital, Fourth Military Medical University. Asthma was diagnosed based on clinical criteria. Patients who had used immunosuppressive drugs in a recent month; had respiratory tract and systemic infections, allergic rhinitis or eczema, or a family history of asthma; or had undergone surgery were excluded from the study. Healthy controls had no history of allergic diseases or respiratory infections. Peripheral blood mononuclear cells (PBMCs) and serum samples were collected after obtaining written informed consent from all participants. The study was approved by the Scientific Research Ethics Committee of the Tangdu Hospital of Fourth Military Medical University (No. K202305-39). Gene expression data of PBMCs and peripheral blood CD4^+^ T and Th2 cells of patients with asthma and normal controls were obtained from the Gene Expression Omnibus (GEO) databases GSE165934, GSE73482, and GSE75011.

### Mice

*Cd226*-floxed mice (*Cd226*^fl/fl^) were created by the Cyagen company (Suzhou, China) [17]. *Cd4*-*Cre* mice were kindly provided by Professor Ning Wu, and *Foxp3*^YFP^-Cre mice were kindly provided by Professor Chen Dong [20]. All mice had the C57BL/6 J background. *Cd226*^fl/fl^ mice were intercrossed with *Cd4*-Cre or *Foxp3*^YFP^-Cre mice to generate T cell-specific *Cd226* KO (*Cd226*^fl/fl^*Cd4*-Cre) or Treg-specific *Cd226* knockout (*Cd226*^fl/fl^*Foxp3*^YFP^-Cre) and its littermate control mice (*Cd226*^fl/fl^). C57BL/6 J mice were procured from the animal center of the Fourth Military Medical University. All mice were housed under specific pathogen-free conditions and were fed with standard laboratory chow and water. All procedures and protocols were approved by the Scientific Research Ethics Committee of the Fourth Military Medical University (No. 20200495). All animal experiments strictly adhere to the international 3 R (Replacement, Reduction, and Refinement) principles to minimize the suffering and the number of experimental animals used, while ensuring statistical validity.

### Asthma induction

OVA (Grade VI, Sigma-Aldrich, St. Louis, MO, USA) immunization and challenge were performed as previously described [21]. Briefly, 8- to 10-week-old mice were randomly divided into PBS and OVA groups. Mice in OVA group were intraperitoneally immunized with 20 μg OVA dissolved in 200 μL phosphate-buffered saline (PBS) and 2 mg aluminum hydroxide (Alum) on days 0 and 14; mice were challenged daily with 1% OVA/PBS aerosolized by nebulizer on days 21–27. Mice in PBS group were immunized and challenged with PBS. Mice were anesthetized 24 h after the last challenge, and their lungs were gently flushed three times via the tracheal tube with 0.7 mL of PBS. The total number of cells in the bronchoalveolar lavage fluid (BALF) was counted using a cell counter (RWD Life Science, Shenzhen, China). BALF was centrifuged at 2000 rpm for 5 min; collected cells were analyzed via flow cytometry (FCM).

### Histological analysis of lung tissues

Lung tissue was removed and fixed in 4% paraformaldehyde, embedded in paraffin, and cut into 4-μm sections. Specimens were xylene deparaffinized and ethanol rehydrated. Sections were then stained with hematoxylin and eosin (H&E), periodic acid–Schiff (PAS), and Picrosirius Red stain. Images were acquired using a digital pathological slide scanner (Grundium Ocus, Finland). Quantification of the inflammation severity, PAS-positive cells (goblet cells), and collagen deposition were performed according to previous studies [22–25].

### Enzyme-linked immunosorbent assay (ELISA)

Mouse total immunoglobulin E (IgE) levels in serum and BALF were determined using a commercialized precoated ELISA kit (Dakewe Biotech, Shenzhen, China). OVA-specific IgE and IgG1 antibody titers were measured according to the manufacturer’s instructions. In brief, a 96-well plate coated with 10 µg/mL OVA was incubated with diluted samples for 2 h. Next, anti-mouse IgE-HRP (Invitrogen) and anti-mouse IgG1-HRP (Invitrogen) were added, and the plate was incubated for 1 h; the plate was washed five times with wash buffer between each step. The optical density was determined by measuring the absorbance at 450/590 nm. To measure serum OVA-specific IgG2a, the 96-well plate was coated with 10 µg/mL OVA at 4 °C overnight and incubated with diluted serum samples for 2 h. Next, they were successively incubated with anti-mouse IgG2–biotin (Invitrogen) and HRP–streptavidin (Yeasen) for 1 h. The plate was washed as mentioned above, and the optical density was measured as described above.

### Quantitative PCR (qPCR)

Total RNA was extracted using TRIGene reagent (GenStar, China) and converted to cDNA using the Hifair II 1st Strand cDNA Synthesis Kit (Yeasen Biotechnology, Shanghai, China). qPCR analysis was performed using the SYBR Master Mix (GenStar) and was quantified via the 2^−ΔΔCt^ method. β-actin was used as the internal control. Transcript levels were normalized to the housekeeping gene β-actin. Primers used are listed in Supplementary Table 1.

### FCM analysis

Human PBMCs were isolated from donors and resuspended in FCM buffer (PBS + 1% fetal calf serum + 0.01% azide), and anti-human CD4 (FITC, RPA‐T4), CXCR3 (APC, G025H7), CRTH2 (PE, BM16), CCR6 (Alexa Fluor 700, G034E3), and CD226 (PE-Cy7, 11A8) (Biolegend, San Diego, CA, USA) were used to determine the expression level of CD226 on T cell subsets. Th1 subsets were identified as CXCR3^+^ CRTH2^−^ CCR6^−^, Th2 subsets as CRTH2^+^ CXCR3^−^, and Th17 subsets as CXCR3^−^ CRTH2^−^ CCR6^+^ [26].

For surface staining, cells from BALF, mouse blood, spleen, and lungs were suspended and incubated with an Fc-blocking antibody (anti-CD16/CD32, Thermo Fisher Scientific, Carlsbad, CA, USA) for 10 min to prevent nonspecific staining and were then stained with fluorochrome-conjugated antibodies at 4 °C for 30 min in the dark. The following antibodies were bought from Thermo Fisher Scientific and BioLegend (fluorochrome and clone numbers are presented in parenthesis): CD45 (Alexa Fluor 700, 30-F11), CD3 (FITC, 145-2C11), CD4 (Pacific Blue, RM4-5), CD8α (PE, 53-6.7), CD69 (FITC, H1.2F3), CD11c (FTIC, N418), CD170/Siglec-F (Super Bright 426, 1RNM44N), Gr-1/Ly6G (APC, RB6-8C5), F4/80 (PE, BM8), NK1.1 (FITC, PK136), B220 (APC, RA3-6B2), CD45.1 (PE-Cy7, A20), and CD45.2 (Alexa Fluor 700, 104).

The nuclear transcription factor Foxp3 staining was performed using a Foxp3/transcription factor staining kit (Thermo Fisher Scientific) according to the manufacturer’s instructions. Data were acquired using a Sony SP6800 Spectral Analyzer (Sony Biotechnology, UK) and the NovoExpress software.

### Apoptosis assay

Cellular apoptosis was detected using the Annexin V/7-AAD apoptosis assay kit with flow cytometry. Cells were separated into four groups: live cells (Annexin V^–^ 7-AAD^–^), cells in early apoptosis (Annexin V^+^ 7-AAD^–^), cells in late apoptosis (Annexin V^+^ 7-AAD^+^), and necrotic cells (Annexin V^−^ 7-AAD^+^). Apoptosis-related protein Caspase-3 activity was determined by detecting cleaved Caspase-3 with FCM analysis.

For in vitro assay, naive CD4^+^ T cells from lymph nodes were isolated with Dynabeads™ Untouched™ Mouse CD4 Cells Kit (Thermo Fisher Scientific). Then, purified CD4^+^ cells were stimulated (5 × 10^5^ cells) with Dynabeads™ mouse T-Activator CD3/CD28 (Thermo Fisher Scientific) for 24 h, or left untreated for 0 and 24 h. For IL-7-mediated cell survival, purified CD4^+^ cells were incubated (5 × 10^5^ cells) in 10 ng/mL recombinant mouse IL-7 media or left untreated for 48 h.

For Akt phosphorylation-mediated cell apoptosis, purified CD4^+^ cells were incubated with 10 μM Akt inhibitor VIII or 4 μg/mL Akt agonist SC79 (MedChemExpress, New Jersey, USA).

### TUNEL analysis

Lung sections were incubated with anti-mouse CD4 antibody (1:3000, Servicebio, Wuhan, China) and corresponding secondary antibody. Then, TUNEL was conducted using a TUNEL Cell Apoptosis Detection Kit (Servicebio) according to the manufacturer’s instructions. Fluorescent images were collected using a fluorescent upright microscope (Nikon Eclipse C1, Tokyo, Japan) and analyzed using CaseViewer version 2.3.

### Western blotting

Protein samples were prepared using RIPA lysate and a protease inhibitor, separated through SDS-PAGE, and transferred to PVDF membranes. Proteins were detected using ECL reagent and Chemidoc imaging system (Bio-Rad) following incubation with primary and secondary antibodies. The following primary antibodies were obtained from Cell Signaling Technology (Danvers, MA, USA) and Proteintech (Chicago, USA): Caspase-3, cleaved Caspase-3, Bax, Akt, p-Akt Ser473, and p-Akt Thr308. The gray density of the protein was determined using Image J software, and the relative expression of the target proteins was quantified as the ratio of their gray intensity to that of Actin.

### Generation of bone marrow chimeras

CD45.1^+^ CD45.2^+^ recipient mice underwent lethal irradiation (8.5 Gy) and were reconstituted with a 1:1 ratio of 5 × 10^6^ wild-type (WT; CD45.1^+^CD45.2^−^) and *Cd226*-knockout (KO; CD45.2^+^CD45.1^−^) bone marrow cells via tail-vein injection. After 6 weeks, the bone marrow chimeric mice were used for the indicated experiments.

### Administration of CD226-Fc fusion protein

Recombinant CD226 fused with human IgG Fc protein (CD226-Fc) was bought from Sino Biological Inc. (Beijing, China); recombinant human IgG Fc (IgG) served as a control in experiments. OVA-sensitized and -challenged mice were treated with CD226-Fc or human IgG at 20 μg/mouse at the indicated time point.

### RNA sequencing (RNA-seq)

Lymphocytes isolated from spleen and lymph nodes from OVA-induced asthmatic *Cd226*^fl/fl^*Cd4*-Cre and *Cd226*^fl/fl^ mice were sorted using FACS (ARIA III sorter; purify >98%) to obtain CD4^+^ T cells. Cells were collected, and total RNA was extracted using TRIzol reagent (Invitrogen, Carlsbad, CA, USA). RNA quality was verified using an Agilent 2100 Bioanalyzer (Agilent Technologies, Palo Alto, CA, USA) and checked using RNase-free agarose gel electrophoresis. RNA-Seq analysis was performed by BGI-Tech using the BGIseq500 platform (BGI, Shenzhen, China). Dr. Tom (https://biosys.bgi.com) network platform was applied to construct and visualize the network.

### Statistical analysis

Statistical data were analyzed using GraphPad Prism 9.0 (GraphPad, La Jolla, CA, USA). Data are presented as mean ± standard error of the mean (SEM). Each scatter represents an independent biological replicate. Kolmogorov–Smirnov test was used to analyze whether the experimental data obeyed normal distribution. *F*-test was used to test the homogeneity of variance. For normal distribution data, independent Student’s *t*-test or one-way analysis of variance was used for comparing different groups. The semiquantitative data of the histopathological parameters were analyzed using the Mann-Whitney *U* nonparametric test. A *P* value of <0.05 indicated statistical significance.

## Results

### CD226 is upregulated in CD4^+^ T cells from patients with asthma

**Fig. 1:**
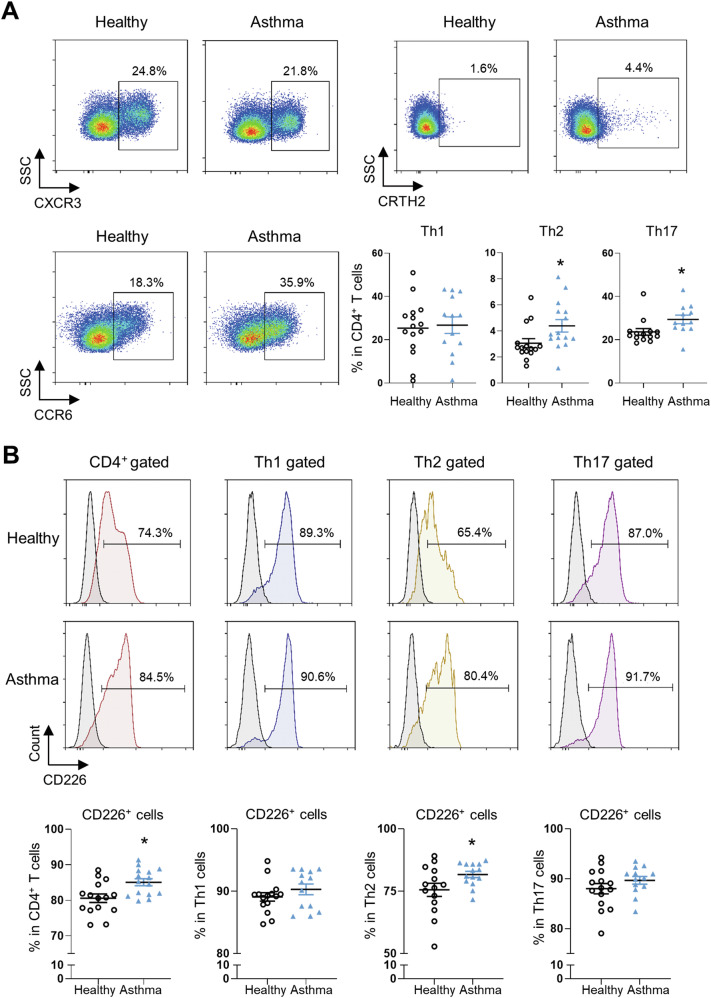
Expression of CD226 is upregulated in CD4^+^ T cells from patients with asthma. **A** Representative FCM histograms depicting the frequency (%) of Th1 (CXCR3^+^CRTH2^−^CCR6^−^), Th2 (CRTH2^+^CXCR3^−^), and Th17 (CXCR3^−^CRTH2^−^CCR6^+^) subsets in CD4^+^ T cells from PBMCs of healthy subjects and patients with asthma. **B** Representative FCM histograms displaying the frequency (%) of CD226^+^ cells in total CD4^+^ T cells, Th1, Th2, and Th17 cells. *P < 0.05 and **P < 0.01 vs. healthy subjects. Each dot represents a different sample. FCM flow cytometry, Th helper T cells, PBMCs peripheral blood mononuclear cells.

Firstly, we conducted FCM analysis to determine the frequencies of Th1, Th2, and Th17 phenotype effector CD4^+^ T cells in asthmatic patients and healthy controls. CD4^+^ T cells were isolated from PBMCs of 15 patients with asthma (mean age: 43.3 years; 7 females) and 15 healthy controls (mean age: 36.2 years; 7 females). Results revealed that the frequencies of circulating Th2 and Th17 cells were higher in patients with asthma than in healthy subjects (Fig. 1A). To study the association between CD226 and asthma, we further detected CD226 levels on these Th cell subsets. Results showed a high CD226 expression in total CD4^+^ T cells, as well as Th1, Th2, and Th17 subsets in human PBMCs. Notably, the expression levels of CD226 on total CD4^+^ T cells and Th2 cells were significantly higher in the asthma group than in the healthy group, while no significant difference was observed between the two groups for Th1 and Th17 cells (Fig. 1B).

Moreover, we downloaded the gene expression profile from the GEO database using data from PBMCs, peripheral blood CD4^+^ T cells and Th2 cells from patients with asthma or healthy subjects. No difference in mRNA expression level of CD226 on PBMCs was observed between the two groups (Supplementary Fig. 1A), whereas the expression of CD226 mRNA of allergen-challenged CD4^+^ cells and Th2 cells was significantly higher in patients with asthma (Supplementary Fig. 1B, C), further confirmed the above FCM results. Allergic responses are triggered and maintained by an imbalance among CD4^+^ Th cells, specifically Th2 cells, therefore, we speculated that CD226 may play a regulatory role in the progression of asthma.

### CD226 deficiency in T cells ameliorates OVA-induced asthma in mice

To assess the function of CD226 for T cells in vivo, we generated mice with a conditional deletion of the *Cd226* gene in CD4^+^ T cells by crossing *Cd4-*Cre mice with mice harboring a floxed *Cd226* allele (*Cd226*^fl/fl^; Supplementary Fig. 2A). Genotyping of mice indicated the CKO of homozygous *Cd226*^fl/fl^ allele and *Cd4‐*Cre (Supplementary Fig. 2B). The knockout efficiency of *Cd226* was determined at the mRNA and protein level using qPCR and FCM analysis, respectively (Supplementary Fig. 2C, D). *Cd226*^fl/fl^*Cd4-*Cre mice were viable and fertile without any appreciable abnormality.

FCM analysis of splenocytes revealed no difference between *Cd226*^fl/fl^*Cd4*-Cre and *Cd226*^fl/fl^ mice in terms of total number of cells, CD3^+^ population, CD3^+^ CD4^+^ T cells, and CD3^+^ CD8^+^ T cells. In addition, the expression of the activation marker CD69 did not differ in *Cd226*-deficient CD4^+^ T cells in the resting state in vivo (Supplementary Figs. 3A. B). These results indicate that the T cell-specific deficiency of CD226 barely affected the development and activation status of T cells during steady state, suggesting that CD226 is dispensable for the development of T cells.

**Fig. 2:**
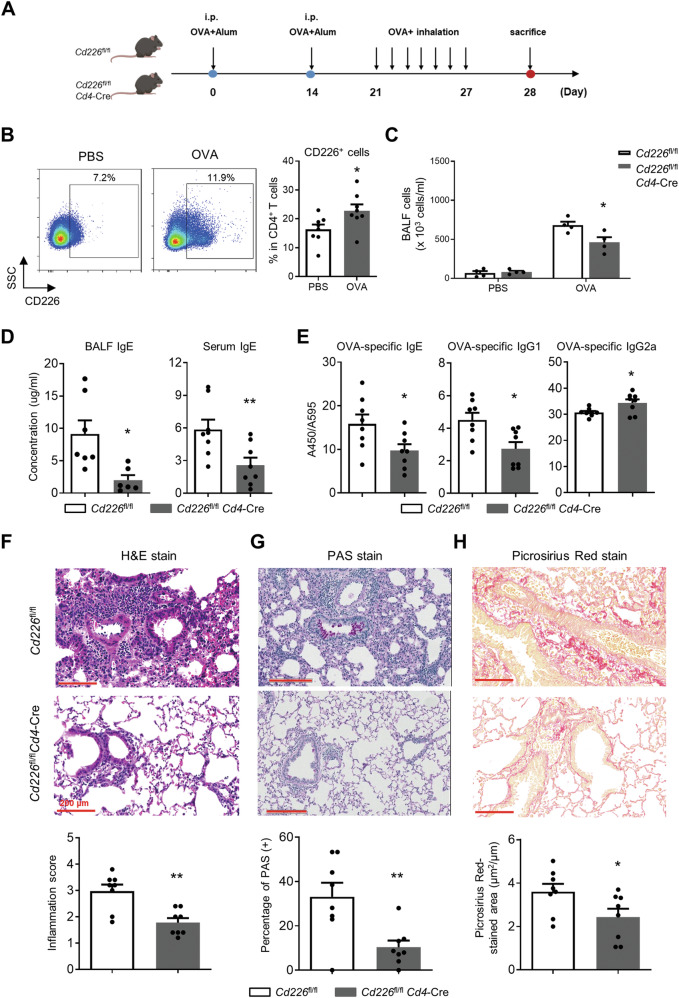
Loss of CD226 in CD4^+^ T cells reduces IgE levels and airway inflammation in an animal model of asthma. **A** Brief description of the protocol of animal sensitization and challenge. On days 0 and 14, mice were sensitized via i.p. injection of the OVA allergen. Mice were then exposed to OVA from day 21 for 7 consecutive days and were sacrificed 24 h after the last challenge. **B** Representative FCM images and frequency (%) of CD226^+^ cells in mice treated with OVA and phosphate-buffered saline (PBS) control; plots were CD4^+^ gated. **C** Total cell number in BALF was counted in *Cd226*^fl/fl^*Cd4*-Cre and *Cd226*^fl/fl^ mice. **D** IgE in BALF and serum were measured. **E** Quantification of anti-OVA-specific IgE, IgG1, and IgG2a antibody isotype in serum of *Cd226*^fl/fl^*Cd4*-Cre and *Cd226*^fl/fl^ mice. **F** Effect of CD226 on lung inflammatory cell infiltration in OVA-induced allergic asthmatic mice assessed by H&E staining. **G** Lung tissue sections were PAS stained to assess goblet cell hyperplasia. **H** Subepithelial collagen deposition and fibrosis were assessed by Picrosirius Red staining, scale bar = 200 μm. Data are presented as mean ± SEM, *P < 0.05, **P < 0.01 vs. *Cd226*^fl/fl^ control. i.p. intraperitoneal, OVA ovalbumin, BALF bronchoalveolar lavage fluid, PAS periodic acid–Schiff, H&E hematoxylin and eosin.

To analyze the roles that CD226 plays in asthma pathogenesis, we used the OVA-induced allergic asthma model with the standard protocol (Fig. 2A). Consistent with the observed trend in human samples, an elevation of CD226 levels on CD4^+^ T cells was observed in asthmatic mice (Fig. 2B). OVA sensitization increased the presence of inflammatory cells in BALF, whereas this was significantly attenuated with CD226 deficiency in T cells (Fig. 2C). The total concentration of serum and BALF IgE was significantly reduced in OVA-challenged *Cd226*^fl/fl^*Cd4*-Cre mice compared with that in *Cd226*^fl/fl^ mice (Fig. 2D). Moreover, the levels of OVA-specific IgE and IgG1 were significantly decreased, whereas IgG2a levels were elevated in *Cd226*^fl/fl^*Cd4*-Cre mice compared with those in *Cd226*^fl/fl^ mice (Fig. 2E). H&E staining of leukocyte infiltration in lung tissues revealed that CD226 deficiency in T cells significantly reduced inflammation and attenuated alveolar wall and pulmonary interstitial edema and congestion (Fig. 2F). Moreover, PAS and Picrsirius Red staining showed that lungs from *Cd226*^*f*l/fl^*Cd4*-Cre mice had significantly diminished OVA-induced goblet cell hyperplasia and progressive diffuse alveolar fibrosis (Fig. 2G, H).

### Loss of CD226 in T cells reduces infiltration of eosinophils in asthmatic mice

**Fig. 3:**
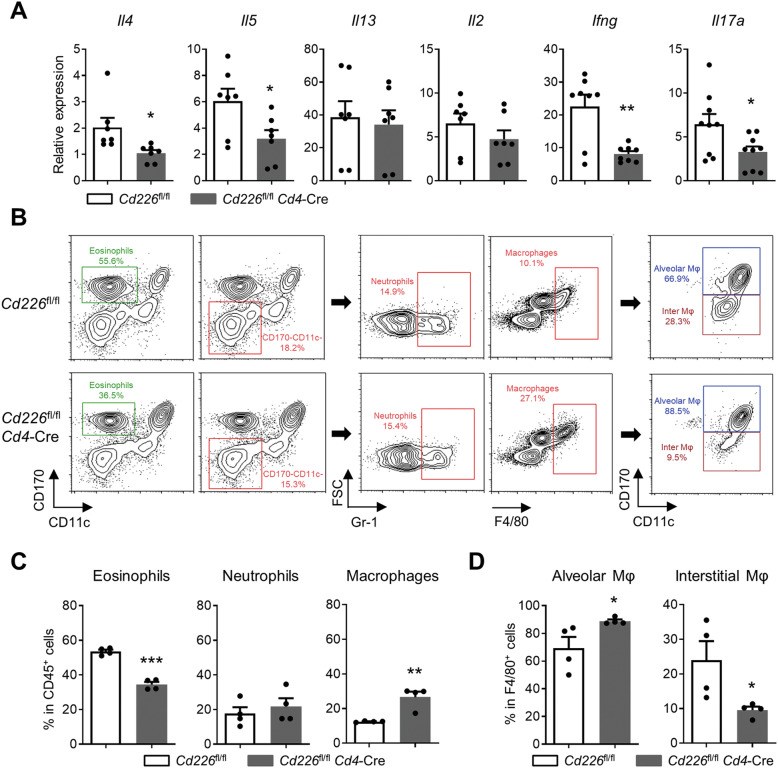
CD226 loss in T cells improves OVA-induced asthma. Mouse lung tissues and BALF of mice were collected immediately after euthanization. **A** qPCR was performed using lung samples to assess the expression of *Il4*, *Il5*, *Il13*, *Il2*, *Ifng* and *Il17a*. **B** Representative FCM histograms depicting the frequency (%) of eosinophils (CD11c^−^CD170^+^), neutrophils (CD11c^−^CD170^–^Gr-1^+^), and macrophages (CD11c^−^CD170^–^F4/80^+^) in BALF from *Cd226*^fl/fl^*Cd4*-Cre and *Cd226*^fl/fl^ mice. Macrophages are further divided into BALF exudate alveolar macrophages (F4/80^+^CD11c^+^CD170^+^) and interstitial macrophages (F4/80^+^CD11c^+^CD170^–^). Displayed FCM plots are CD45^+^ gated. **C** Quantitative data of FCM analysis for eosinophils, neutrophils, and macrophages in total CD45^+^ leukocytes. **D** Quantitative data of FCM analysis for exudate alveolar and interstitial macrophages in BALF. All data are presented as mean ± SEM; *P < 0.05, **P < 0.01, ***P < 0.001 vs. *Cd226*^fl/fl^ control. OVA ovalbumin, BALF bronchoalveolar lavage fluid, FCM flow cytometry, Mφ macrophage.

Allergic asthma is associated with dysregulation of cytokine expression. After OVA induction, we evaluated the mRNA expression of Th1 cytokines (IL-2 and IFN-γ), Th2 cytokines (IL-4, IL-5 and IL-13), and Th17 cytokine IL-17a in the lung tissues of the mice in the two groups. The expression of *Il4*, *Il5*, *Ifng*, and *Il17a* mRNA in the lungs was considerably downregulated in *Cd226*^fl/fl^*Cd4*-Cre mice compared with that in *Cd226*^fl/fl^ mice (Fig. 3A). Therefore, CD226 loss in T cells reduced the OVA-induced inflammatory cytokines in lungs.

FCM analysis was performed to evaluate the leukocytes entering the lungs, including eosinophils, neutrophils, and macrophages in BALF after the OVA challenge. The results showed a significant reduction in the number of eosinophils. The recruitment and accumulation of these cells in BALF is the hallmark of allergic asthma and a marker of disease severity. Meanwhile, there is a significantly higher frequency of macrophages in *Cd226*^fl/fl^*Cd4*-Cre mice than that in *Cd226*^fl/fl^ mice (Fig. 3B, C). Identification of exudate alveolar and interstitial macrophages showed that the frequency of alveolar cells increased, whereas that of interstitial macrophages decreased because of the CD226 deficiency in T cells (Fig. 3B, D). According to previous studies, interstitial macrophages are derived from circulating monocytes, whereas alveolar macrophages are highly specialized mononuclear phagocytic cells located in the alveolar space, which predominantly express an M2 phenotype [27–29].

### CD226 influences asthma development via a Treg-independent mechanism

Activated CD4^+^ T cells proliferate and differentiate into effector Th cells or Treg cells. Treg cells have a marked negative regulatory immunologic function and play a crucial role in maintaining immune homeostasis in asthma. Therefore, we evaluated whether the effect of CD226 on the asthmatic mice was mediated by Foxp3^+^ Treg cells. FCM analysis showed that the percentage of Foxp3^+^ Treg cells did not differ between *Cd226*^fl/fl^ and *Cd226*^fl/fl^*Cd4*-Cre mice upon OVA treatment (Supplementary Fig. 4A). We further generated Treg-specific *Cd226* CKO mice (*Cd226*^fl/fl^ *Foxp3*^YFP^-Cre). HE and PAS staining revealed that CD226 deficiency in Treg cells did not affect lung inflammation or goblet cell hyperplasia or the total number of infiltrated cells in BALF (Supplementary Fig. 4B–D). Further FCM analysis demonstrated that the frequency of eosinophils and macrophages (alveolar and interstitial macrophages) in BALF did not differ between *Cd226*^fl/fl^ *Foxp3*^YFP^-Cre mice or littermate control mice (Supplementary Fig. 4E–G). Thus, the effects of CD226 on the asthmatic mice may be mainly mediated by effector Th cells but not by Treg cells.

### CD226 may affect apoptosis of CD4^+^ T cells

**Fig. 4:**
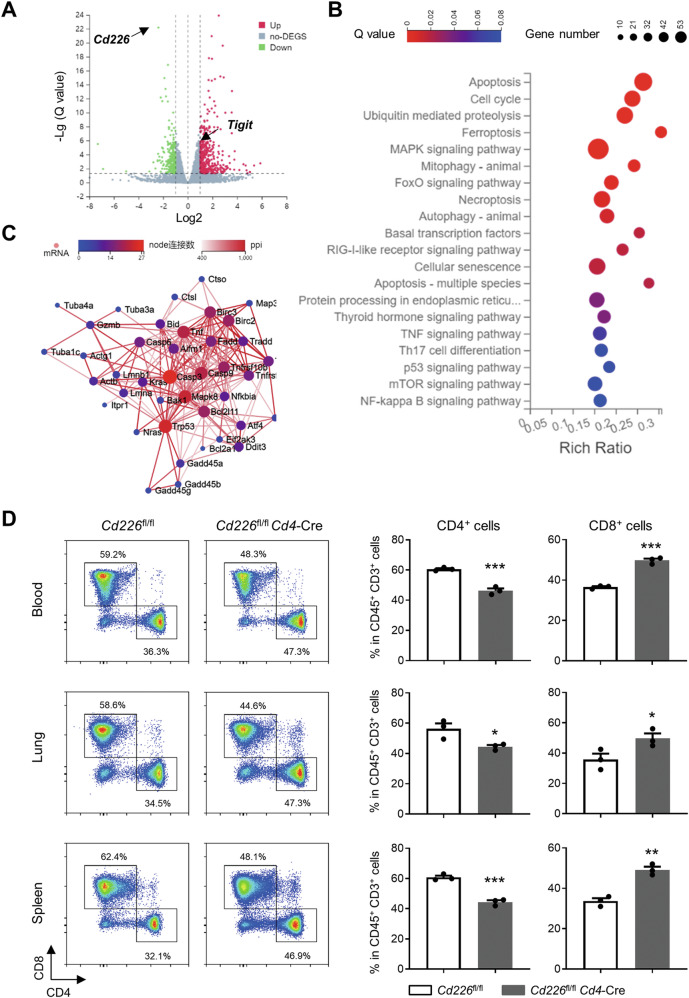
RNA-seq analysis of CD4^+^ T cells from *Cd226*^fl/fl^*Cd4*-Cre and *Cd226*^fl/fl^ mice. **A** Volcano plot showed DEGs between CD4^+^ T cells from *Cd226*^fl/fl^*Cd4*-Cre and *Cd226*^fl/fl^. **B** KEGG pathway enrichment of DEGs. **C** PPI networks were performed to analysis the DEGs annotated to the apoptosis pathway. For the RNA-seq experiment, n = 3 replicates per group. **D** Representative FCM images and quantification of CD4^+^ and CD8^+^ cells among CD45^+^ CD3^+^ cells in the peripheral blood, lung, and spleen samples from *Cd226*^fl/fl^*Cd4*-Cre and *Cd226*^fl/fl^ asthmatic mice. Data are presented as mean ± SEM; *P < 0.05, **P < 0.01, ***P < 0.001. DEGs differentially expressed genes, OVA ovalbumin, KEGG Kyoto encyclopedia of genes and genomes, PPI protein–protein interaction networks, FCM flow cytometry.

To reveal the underlying mechanisms, we conducted RNA-seq analysis of the CD4^+^ T cells from *Cd226*^fl/fl^*Cd4*-Cre and *Cd226*^fl/fl^ mice with OVA induction. This identified 994 downregulated and 1081 upregulated differentially expressed genes (DEGs) between these mice after asthma induction (Q value <0.05). Among them, the mRNA level of CD226 significantly decreased, whereas that of its counterpart inhibitory molecule TIGIT significantly increased (Fig. 4A). Furthermore, KEGG pathway analysis showed significant enrichment of DEGs between the OVA-immunized mice in apoptosis, cell cycle, ubiquitin-mediated proteolysis, ferroptosis, MAPK-signaling pathway, mitophagy, and other pathways, among which apoptosis was the most highly enriched pathway containing 41 DEGs (Fig. 4B). Subsequently, the differential proteins annotated to the apoptosis pathway were analyzed via protein–protein interaction (PPI) networks, which identified *Casp3*, *Mapk8*, *Trp53*, *Casp9*, and *Tnf* as the most prevalent hub genes (Fig. 4C). Then, lymphocytes from peripheral blood, lungs, and spleen of *Cd226*^fl/fl^*Cd4*-Cre and *Cd226*^fl/fl^ asthmatic mice were collected and analyzed. FCM analysis revealed a shift in CD45^+^ CD3^+^ T cell populations. Specifically, the proportion of CD4^+^ cells decreased, whereas that of CD8^+^ cells increased in T cells (Fig. 4D). These results suggest that the deletion of CD226 may trigger apoptosis in CD4^+^ T cells in patients with allergic asthma.

### CD226 deficiency promotes late apoptosis of CD4^+^ T cell

**Fig. 5:**
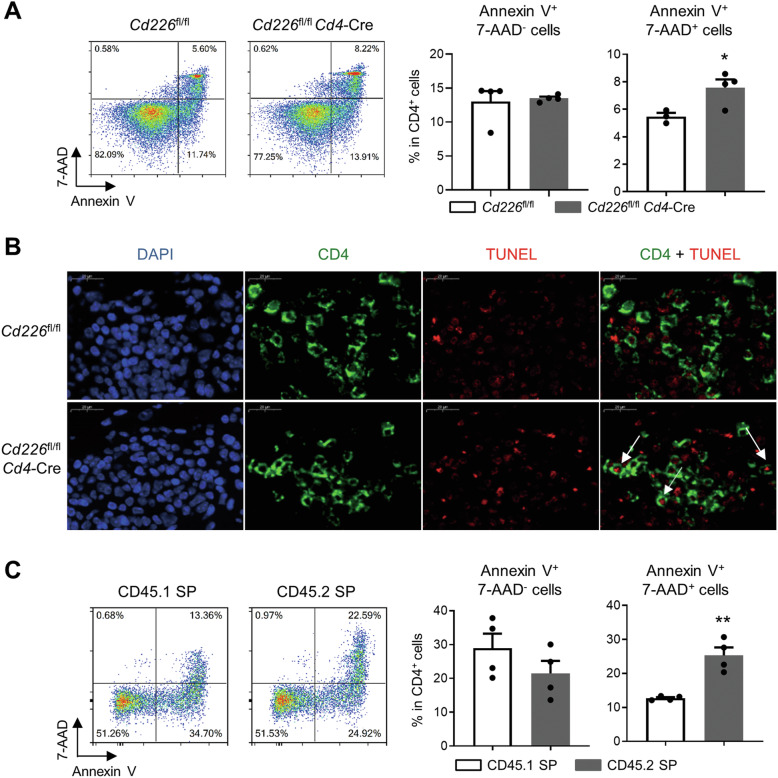
CD226 deficiency promotes late apoptosis of CD4^+^ T cells. **A** The apoptosis of CD4^+^ cells from the spleen of *Cd226*^fl/fl^*Cd4*-Cre and *Cd226*^fl/fl^ asthmatic mice were detected via Annexin V and 7-AAD staining and FCM analysis. **B** Representative fluorescent images of apoptotic CD4^+^ T cells in lung tissue from asthmatic mice; scale bar = 20 μm. **C** Annexin V^+^ 7-AAD^−^ and Annexin V^+^ 7-AAD^+^ cells in CD45.1 SP and CD45.2 SP CD4^+^ cells evaluated via FCM. Data are presented as mean ± SEM; *P < 0.05, **P < 0.01. SP single positive, FCM flow cytometry.

Next, we detected the apoptosis of CD4^+^ T cells after conditionally knocking out the CD226 gene in asthmatic mice. The Annexin V^+^ 7-AAD^+^ late apoptotic CD4^+^ T cells were significantly increased in *Cd226*^fl/fl^*Cd4*-Cre mice compared with *Cd226*^fl/fl^ mice, whereas there was no difference in Annexin V^+^ 7-AAD^−^early apoptotic cells (Fig. 5A). To further characterize T cells undergoing apoptosis, the TUNEL technique was combined with CD4 cell surface staining. As shown in Fig. 5B, the deletion of CD226 in CD4^+^ T cells caused a significant increase in TUNEL-positive cells in the lungs of asthmatic mice, indicating that deletion of CD226 promotes CD4^+^ T cell apoptosis.

Moreover, to determine whether the increased frequency of apoptotic CD4^+^ T cells in *Cd226-*deletion mice was cell intrinsic, we generated bone marrow chimeric mice by adoptively transferring WT and CD226 KO mixed bone marrow cells into recipient mice. In this model, WT donor-derived cells were identified as CD45.1^+^ CD45.2^–^ cells, whereas CD226 KO donor-derived cells were identified as CD45.2^+^ CD45.1^–^ cells. The proportions of CD4^+^, CD8^+^, NK cells, and B cells among CD45.1 single positive (SP) and CD45.2 SP splenocytes were comparable, *Cd226*-deletion had minimal effect on T cell development in the steady state (Supplementary Fig. 5). Furthermore, the proportion of Annexin V^+^ 7-AAD^+^ late apoptotic cells in CD45.2 SP CD4^+^ T cells was significantly higher than that in CD45.1 SP cells (Fig. 5C), validating the finding that CD226 deficiency accelerated the late apoptotic of CD4^+^ T cells.

### CD226 deficiency promotes Caspase-3 activity in CD4^+^ T cells

**Fig. 6:**
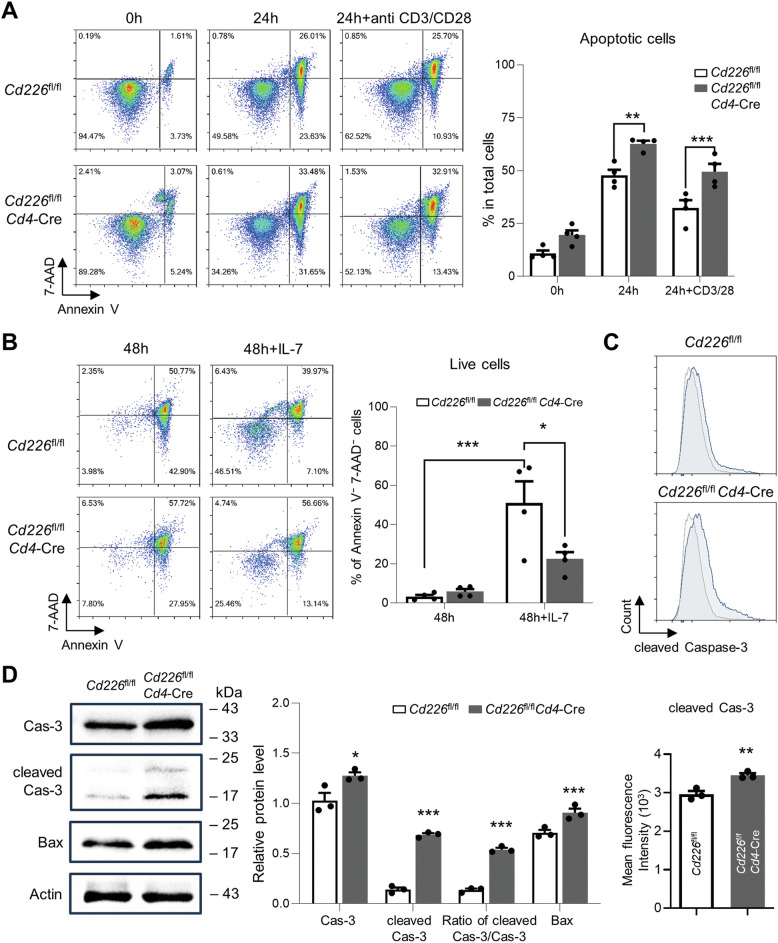
CD226 deficiency promotes activation of Caspase-3 in CD4^+^ T cells. **A** CD4^+^ cells were purified from mouse lymph nodes and were stimulated with anti-CD3/CD28 Dynabeads or left untreated for 0 and 24 h. Annexin V/7-AAD staining and FCM analysis were performed to assess cell apoptosis. **B** Purified CD4^+^ cells were cultured with or without IL-7 for 48 h. Cells were collected and subjected to Annexin V/7-AAD staining and FCM analysis. **C** Caspase-3 activity assay. CD4^+^ cells isolated from the lymph nodes of mice were incubated with anti-CD3/CD28 Dynabeads for 24 h. The level of cleaved Caspase-3 in CD4^+^ T cells was assessed via FCM analysis. The mean fluorescence intensity of cleaved Caspase-3 was quantified and shown in the lower panel. **D** The level of Caspase-3, cleaved Caspase-3, and Bax proteins in CD4^+^ T cells was analyzed via western blotting. Data are shown as mean ± SEM; *P < 0.05, **P < 0.01, ***P < 0.001. Cas-3 Caspase-3.

To confirm these findings obtained from in vivo, we further studied the role of CD226 on CD4^+^ cells apoptosis in ex vivo experiments. CD4^+^ cell apoptosis was significantly increased in the *Cd226*^fl/fl^*Cd4*-Cre group compared with that in the *Cd226*^fl/fl^ group following treatment with or without anti-CD3/CD28 antibodies treatment for 24 h, suggesting that CD226 deficiency increased the CD4^+^ cell apoptosis in both natural apoptotic and T cell receptor (TCR) activation states (Fig. 6A).

IL-7 is a crucial pro-survival cytokine for T cells, therefore, we tested whether deletion of *Cd226* could suppress T cells survival in the presence of IL-7. As shown in Fig. 6B, after 48 h culturinge, the majority of cells tend to undergo apoptosis with few live cells (7-AAD^−^ Annexin V^−^) without IL-7; however, the presence of IL-7 significantly increased the proportion of live cells. Notably, live CD4^+^ T cells significantly reduced by deletion of *Cd226* after 48 h incubation with IL-7 (22.4 ± 3.5 % alive cells in the *Cd226*^fl/fl^*Cd4*-Cre group compared to 51.0 ± 11.0 % in the control). These data further indicate that CD226 can be an important contributing anti-apoptotic factor.

Above RNA-seq and PPI networks results indicated that Caspase-3 is the hub gene, and it may be crucial for CD226 to modulate CD4^+^ T cells function. FCM analysis showed that the increased CD4^+^ cell apoptosis was accompanied by an elevated level of intracellular Caspase-3 in *Cd226*^fl/fl^*Cd4*-Cre group; and western blotting showed significantly upregulated pro-apoptotic proteins, cleaved Caspase-3 and Bax, as well as an increased ratio of cleaved Caspase-3/Caspase-3 in *Cd226*^fl/fl^*Cd4*-Cre group (Fig. 6C, D), indicating the involvement of intrinsic apoptotic pathway in CD226-mediated cell apoptosis.

### CD226 modulates apoptosis of CD4^+^ T cells through an Akt-dependent pathway

**Fig. 7:**
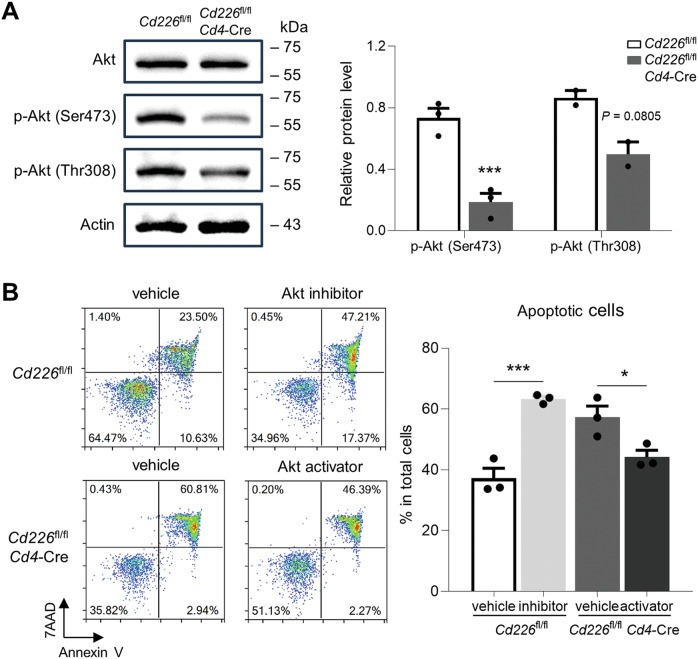
CD226 regulates CD4^+^ T cell apoptosis via an Akt-dependent pathway. **A** CD4^+^ cells were purified from mouse lymph nodes and were incubated with anti-CD3/CD28 Dynabeads for 24 h. The level of Akt, phosphorylated Akt (Ser473), and phosphorylated Akt (Thr308) in the CD4^+^ T cell was detected via western blotting. **B** Purified CD4^+^ cells were incubated with anti-CD3/CD28 Dynabeads and Akt inhibitor VIII or Akt agonist SC79 for 24 h; cell apoptosis was detected via FCM. Data are presented as mean ± SEM; *P < 0.05, ***P < 0.001.

The Akt pathway serves as a pivotal signaling cascade in the regulation of cell survival and anti-apoptosis. Upon TCR activation, downstream components including Akt and MAPK undergo phosphorylation [30, 31]. According to the current knowledge [32, 33], both Ser473 and Thr308 phosphorylation sites of Akt are related to cell apoptosis. Akt is also an important downstream signaling molecule in the CD226/TIGIT axis [11]. Western blot analysis showed a significant reduction in the phosphorylation level of Akt at the Ser473 site in CD4^+^ T cells from *Cd226*^fl/fl^*Cd4*-Cre mice (Fig. 7A). Treatment with Akt inhibitor VIII promoted apoptosis in CD4^+^ T cells from the *Cd226*^fl/fl^ control group, while CD226-deficient CD4^+^ T cells exhibited higher levels of apoptosis, which were significantly reversed by the Akt agonist SC79 (Fig. 7B).

### Therapeutic effects of CD226-Fc recombinant protein on OVA-induced allergic asthma

**Fig. 8:**
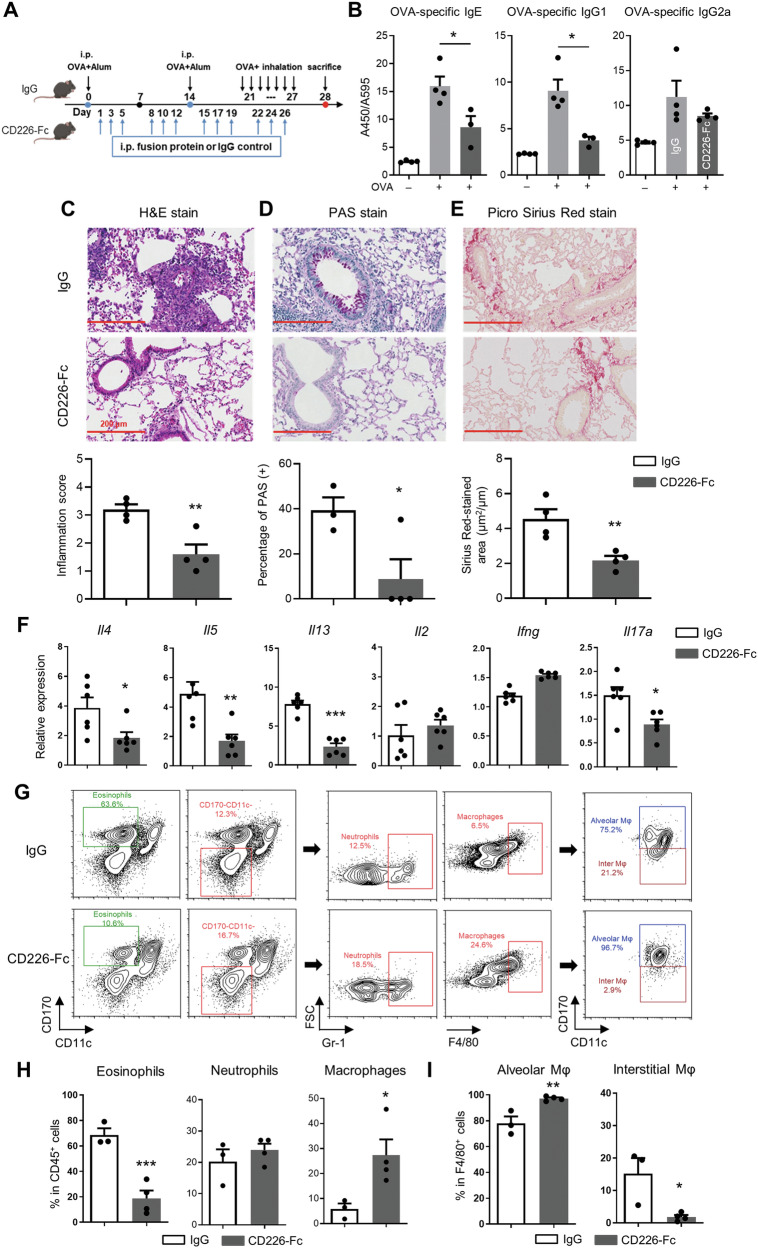
Recombinant protein CD226-Fc ameliorates lung tissue damage and inflammation in asthmatic mice. **A** Experimental protocol of sensitization, OVA challenge, and administration with CD226-Fc in an asthma model. Mice were administered via intraperitoneal injection (i.p.) with CD226-Fc or human IgG at a dose of 20 μg/mouse at the indicated time point. **B** Quantification of anti-OVA-specific IgE, IgG1, and IgG2a antibody isotypes in sera via the corresponding commercial kits. **C** H&E staining indicates the therapeutic effect of CD226-Fc on lung inflammatory cell infiltration in OVA-induced allergic asthmatic mice. **D** Lung tissue sections were subjected to PAS staining to assess goblet cell hyperplasia. **E** Collagen deposition and fibrosis was determined by Picrosirius Red stain. Scale bar = 200 μm. **F** Mouse lung tissues and BALF were collected immediately after euthanization. qPCR was performed for lung samples to assess the levels of *Il4*, *Il5*, *Il13*, *Il2*, *Ifng*, and *Il17a*. **G** FCM analysis of OVA-immunized mice treated with CD226-Fc. Representative FCM histograms depicting the frequency of eosinophils (CD11c^−^CD170^+^), neutrophils (CD11c^−^CD170^–^Gr-1^+^), and macrophages (CD11c^−^CD170^–^F4/80^+^) in BALF from CD226-Fc- or IgG-treated mice. Macrophages were divided into BALF exudate alveolar (F4/80^+^CD11c^+^CD170^+^) and interstitial (F4/80^+^CD11c^+^CD170^–^) macrophages. Displayed FCM plots are CD45^+^ gated. **H** Quantitative data of FCM analysis for eosinophils, neutrophils, and macrophages in total CD45^+^ leukocytes. **I** Quantitative data of FCM analysis for exudate alveolar and interstitial macrophages in BALF. Data are presented as mean ± SEM; *P < 0.05, **P < 0.01, ***P < 0.001 vs. IgG control. BALF bronchoalveolar lavage fluid, i.p. intraperitoneal, OVA ovalbumin, PAS periodic acid–Schiff, H&E hematoxylin and eosin, IgG immunoglobulin G, CD226-Fc recombinant CD226 fused to human IgG Fc protein, FCM flow cytometry, Mφ macrophage.

Finally, we investigated the effects of the recombinant CD226 fusion protein (CD226-Fc) on airway inflammation and remodeling in mice with allergic asthma. C57BL/6J WT mice with OVA-induced asthma were administered intraperitoneally with CD226-Fc fusion protein or IgG (Fig. 8A). Notably, serum levels of OVA-specific IgE and IgG1 were significantly decreased in CD226-Fc-treated mice (Fig. 8B). H&E, PAS, and Picrosirius Red staining were performed to examine the effects of CD226 blockage on lung tissues. The results demonstrated a reduction in pathological abnormalities, such as perivascular infiltration of inflammatory cells, narrowing of the lumen, and thickening of lung mucosa, in the OVA-induced asthmatic mice following CD226-Fc administration (Fig. 8C–E).

The mRNA levels of Th2 and Th17-related cytokines, *Il4, Il5*, *Il13*, and *Il17a* in the lungs were significantly lower in CD226-Fc-treated mice than in the IgG-treated mice (Fig. 8F). Additionally, CD226-Fc administration reduced the frequency of eosinophil, whereas the percentage of macrophages was significantly higher in CD226-Fc-treated mice than that in the IgG-treated mice in BALF evaluated by FCM (Fig. 8G, H). Furthermore, the percentage of alveolar macrophages increased, whereas that of interstitial macrophages decreased after CD226-Fc administration (Fig. 8G, I).

## Discussion

Asthma is pathologically viewed as a chronic inflammatory disease of the lung and is characterized by airway hyperresponsiveness, inflammation, and remodeling [34]. In general, asthma is caused by Th-biased responses with an increased number of inflammatory cells in the airway, and Th2 cytokines further induce an inflammatory cascade that comprises allergen-specific IgE production, mast cell activation, eosinophil recruitment, and airway hyperresponsiveness [35]. Herein, in addition to the attenuated Th2 response, we observed a reduction in Th17 cytokines in the lungs of both *Cd226*^fl/fl^*Cd4*-Cre and CD226-Fc-treated mice with OVA-induced asthma. IL-17a is a cytokine secreted by CD4^+^ Th17, γδT cells, NK cells, and innate lymphoid cells [36]. The expression of IL-17a is increased in the BALF of patients with moderate-to-severe asthma, and the expression of the mucin gene is increased in human airway epithelial cells [37]. We observed that the level of IL-17a was significantly reduced in the lung tissues of *Cd226*^fl/fl^*Cd4*-Cre mice and CD226-Fc-treated mice with OVA-induced asthma; however, the level of IL-17a in mediastinal lymph nodes was comparable in *Cd226*^fl/fl^*Cd4*-Cre (CD226-Fc-treated) and *Cd226*^fl/fl^ (IgG-treated) mice (data not shown). Therefore, we considered that the antiallergic effects observed in *Cd226*^fl/fl^*Cd4*-Cre and CD226-Fc-treated mice may be partially due to the inhibition of Th2/Th17 responses in asthmatic lungs, and CD226-deficient CD4^+^ T cells could trigger immune responses within affected tissues under pathological conditions.

Subsequent RNA-seq analysis and KEGG pathway enrichment indicated that CD226 modulated CD4^+^ T cells apoptosis. CD4^+^ T cells isolated from asthmatic mice and bone marrow chimeric mice both proved that loss of CD226 significantly increases late apoptosis. The in vitro experiment found that CD226 deficiency leads to cell apoptosis with or without TCR activation. Additionally, IL-7 is a key factor for the survival and homeostatic peripheral expansion of T cells, and also has an important anti-apoptotic functions [38, 39]. The pro-survival effect of IL-7 on T cells is associated with the inhibition of Caspase-3 and the increase of anti-apoptotic molecule Bcl-2 [40]. Here, another experiment also proved that loss of CD226 reduced the T cells' survival ability in the presence of IL-7. Meanwhile, we found that the deletion of CD226 increased CD4^+^ cell apoptosis and enhanced the activity of Caspase-3. Caspase-3 is acknowledged as the hallmark of apoptosis and is cleaved and activated at the end of the apoptotic cascade [41]. Caspase-3 activation leads to late-stage apoptosis, and apoptotic cell death in this stage is inevitable [42]. Here, CD226 deficiency mainly induced late apoptosis against CD4^+^ T cells, we speculated that attributed to the regulation of Caspase-3 activity by CD226.

The Akt protein, also referred to as protein kinase B, is a member of the AGC kinase subfamily and is categorized into three subtypes in mammals: Akt1, Akt2, and Akt3. Akt plays an important role in cell survival and apoptosis, it could inhibit T cell apoptosis; inhibition of Akt also induced Caspase-3-dependent cell apoptosis in T lymphocytes [43, 44]. Here, we confirmed that the Akt inhibitor could promote CD4^+^ T cell apoptosis and that CD226-mediated CD4^+^ T cell apoptosis can be inhibited by Akt agonist, indicating that the CD226 deficiency promoted CD4^+^ T cell apoptosis via Akt inhibition.

Meanwhile, we found an increase in its counterpart inhibitory molecule, TIGIT, in CD226-deleted CD4^+^ T cells. TIGIT, CTLA-4, PD-1, T cell immunoglobulin 3 (Tim3), and lymphocyte activation gene 3 (LAG3) are the most commonly targeted checkpoints for cancer immunotherapy [45]. The expression levels of TIGIT and CD226 depend on the T cell subset and activation level, and an increasing number of studies have demonstrated the importance of the TIGIT/CD226 axis in tumors and autoimmune diseases [9, 46, 47]. In this study, the impact of elevated levels of TIGIT cannot be ruled out, the role of TIGIT in T cell apoptosis and allergic diseases, and whether TIGIT interacts with CD226 and affects CD226 function, should be explored in future studies.

Notably, administering CD226-Fc in vivo produced a slightly higher therapeutic effect with *Cd226*^fl/fl^*Cd4*-Cre mice among OVA-induced mice with asthma, as evidenced by a lower percentage of eosinophils in BALF and lower levels of OVA-specific IgE and IgG1 in serum. Therefore, in addition to T cells, the CD226 signal blocked in other immune cells may be involved in improving asthma. Previously, we demonstrated the regulatory impact of CD226 on ILC2s in nasal mucosa during allergic rhinitis [48]. Furthermore, Sakano et al. recently revealed the regulatory impact of CD226 on the effector function of ILC2s in airway hyperresponsiveness [19]. In addition, Bachelet et al. indicated that other effector cells (mast cells and eosinophils) in asthma also express CD226 and its ligand CD112 [49].

This study demonstrated the regulatory role of CD226 in allergic asthma by modulating the apoptosis of CD4^+^ T cells. Blocking CD226 signaling showed an obvious therapeutic effect on allergic asthma in mice. However, further investigation focusing on the mechanisms of CD226 is warranted to elucidate its immunomodulatory activities in T cells. This would further support CD226 inhibition as a basis for targeted therapeutics against allergic airway diseases.

## Supplementary information

Supplementary Information

Supplementary Information-WB original images

## Data Availability

The RNA-seq analysis data that support the findings of this study are openly available in China National Center for Bioinformation (CNCB) Datasets at https://www.cncb.ac.cn/ (BioProject number PRJCA028595, OMIX ID OMIX007005).

## References

[1] Cloutier MM, Dixon AE, Krishnan JA, Lemanske RF Jr, Pace W, Schatz M. Managing Asthma In Adolescents And Adults: 2020 Asthma Guideline Update From the National Asthma Education and Prevention Program. JAMA. 2020;324:2301–17. 33270095 10.1001/jama.2020.21974

[2] Song J, Kang J, Lin B, Li J, Zhu Y, Du J, et al. Mediating role of TRPV1 ion channels in the co-exposure to PM_2.5_ and Formaldehyde of Balb/c mice asthma Model. Sci Rep. 2017;7:11926. 28931832 10.1038/s41598-017-11833-6 PMC5607312

[3] Bousquet J, Mantzouranis E, Cruz AA, Ait-Khaled N, Baena-Cagnani CE, Bleecker ER, et al. Uniform definition of asthma severity, control, and exacerbations: document presented for the World Health Organization Consultation on Severe Asthma. J Allergy Clin Immunol. 2010;126:926–38. 20926125 10.1016/j.jaci.2010.07.019

[4] Lindell DM, Berlin AA, Schaller MA, Lukacs NW. B cell antigen presentation promotes Th2 responses and immunopathology during chronic allergic lung disease. PloS one. 2008;3:e3129. 18769622 10.1371/journal.pone.0003129 PMC2518863

[5] Lloyd CM, Hawrylowicz CM. Regulatory T cells in asthma. Immunity. 2009;31:438–49. 19766086 10.1016/j.immuni.2009.08.007 PMC3385348

[6] Israel E, Reddel HK. Severe and difficult-to-treat asthma in adults. N. Engl J Med. 2017;377:965–76. 28877019 10.1056/NEJMra1608969

[7] Shibuya A, Campbell D, Hannum C, Yssel H, Franz-Bacon K, McClanahan T, et al. DNAM-1, a novel adhesion molecule involved in the cytolytic function of T lymphocytes. Immunity. 1996;4:573–81. 8673704 10.1016/s1074-7613(00)70060-4

[8] Burns GF, Triglia T, Werkmeister JA, Begley CG, Boyd AW. TLiSA1, a human T lineage-specific activation antigen involved in the differentiation of cytotoxic T lymphocytes and anomalous killer cells from their precursors. J Exp Med. 1985;161:1063–78. 2580933 10.1084/jem.161.5.1063 PMC2187589

[9] Shibuya A, Shibuya K. DNAM-1 versus TIGIT: competitive roles in tumor immunity and inflammatory responses. Int Immunol. 2021;33:687–92. 34694361 10.1093/intimm/dxab085

[10] Yasutomi M, Christiaansen AF, Imai N, Martin-Orozco N, Forst CV, Chen G, et al. CD226 and TIGIT cooperate in the differentiation and maturation of human Tfh cells. Front Immunol. 2022;13:840457. 35273617 10.3389/fimmu.2022.840457 PMC8902812

[11] Chiang EY, Mellman I. TIGIT-CD226-PVR axis: advancing immune checkpoint blockade for cancer immunotherapy. J Immunother Cancer. 2022;10:e004711. 35379739 10.1136/jitc-2022-004711 PMC8981293

[12] Zhang Z, Wu N, Lu Y, Davidson D, Colonna M, Veillette A. DNAM-1 controls NK cell activation via an ITT-like motif. J Exp Med. 2015;212:2165–82. 26552706 10.1084/jem.20150792 PMC4647266

[13] Jin HS, Ko M, Choi DS, Kim JH, Lee DH, Kang SH, et al. CD226(hi)CD8(+) T cells are a prerequisite for Anti-TIGIT immunotherapy. Cancer Immunol Res. 2020;8:912–25. 32265229 10.1158/2326-6066.CIR-19-0877

[14] Shapiro MR, Yeh WI, Longfield JR, Gallagher J, Infante CM, Wellford S, et al. CD226 deletion reduces Type 1 diabetes in the NOD mouse by impairing thymocyte development and peripheral T cell activation. Front Immunol. 2020;11:2180. 33013915 10.3389/fimmu.2020.02180 PMC7500101

[15] Fang L, Zhang X, Miao J, Zhao F, Yang K, Zhuang R, et al. Expression of CD226 antagonizes apoptotic cell death in murine thymocytes. J Immunol. 2009;182:5453–60. 19380793 10.4049/jimmunol.0803090

[16] Piedavent-Salomon M, Willing A, Engler JB, Steinbach K, Bauer S, Eggert B, et al. Multiple sclerosis associated genetic variants of CD226 impair regulatory T-cell function. Brain. 2015;138:3263–74. 26359290 10.1093/brain/awv256

[17] Mu Y, Zhang J, Liu Y, Ma J, Jiang D, Zhang X, et al. CD226 deficiency on regulatory T cells aggravates renal fibrosis via up-regulation of Th2 cytokines through miR-340. J Leukoc Biol. 2020;107:573–87. 31802539 10.1002/JLB.2MA1119-174RR

[18] Liu T, Zhang D, Zhang Y, Xu X, Zhou B, Fang L, et al. Blocking CD226 promotes allogeneic transplant immune tolerance and improves skin graft survival by increasing the frequency of regulatory T cells in a murine model. Cell Physiol Biochem. 2018;45:2338–50. 29550821 10.1159/000488182

[19] Sakano Y, Sakano K, Hurrell BP, Helou DG, Shafiei-Jahani P, Kazemi MH, et al. Blocking CD226 regulates type 2 innate lymphoid cell effector function and alleviates airway hyperreactivity. J Allergy Clin Immunol. 2024;153:1406–1422.e6. 10.1016/j.jaci.2024.01.003 38244725

[20] Rubtsov YP, Rasmussen JP, Chi EY, Fontenot J, Castelli L, Ye X, et al. Regulatory T cell-derived interleukin-10 limits inflammation at environmental interfaces. Immunity. 2008;28:546–58. 18387831 10.1016/j.immuni.2008.02.017

[21] Bryce PJ, Mathias CB, Harrison KL, Watanabe T, Geha RS, Oettgen HC. The H1 histamine receptor regulates allergic lung responses. J Clin Investig. 2006;116:1624–32. 16680192 10.1172/JCI26150 PMC1448167

[22] Wang Z, Li L, Wang C, Piao Y, Jiang J, Li L, et al. Recombinant Pyrin Domain protein attenuates airway inflammation and alleviates Epithelial-Mesenchymal transition by inhibiting crosstalk between TGFbeta1 and Notch1 signaling in chronic asthmatic mice. Front Physiol. 2020;11:559470. 33192556 10.3389/fphys.2020.559470 PMC7645102

[23] Kammala AK, Bahal D, Yang C, Panettieri RA Jr, Das R, Subramanian H. Na(+)/H(+) exchanger regulatory Factor 1 mediates the pathogenesis of airway inflammation in a murine model of house dust mite-induced asthma. J Immunol. 2021;206:2301–11. 33952618 10.4049/jimmunol.2001199 PMC8113128

[24] Cho JY, Miller M, Baek KJ, Han JW, Nayar J, Lee SY, et al. Inhibition of airway remodeling in IL-5-deficient mice. J Clin Invest. 2004;113:551–60. 14966564 10.1172/JCI19133 PMC338264

[25] Kujur W, Gurram RK, Haleem N, Maurya SK, Agrewala JN. Caerulomycin A inhibits Th2 cell activity: a possible role in the management of asthma. Sci Rep. 2015;5:15396. 26481184 10.1038/srep15396 PMC4612543

[26] Ercolano G, Wyss T, Salome B, Romero P, Trabanelli S, Jandus C. Distinct and shared gene expression for human innate versus adaptive helper lymphoid cells. J Leukoc Biol. 2020;108:723–37. 32017245 10.1002/JLB.5MA0120-209R PMC7496918

[27] Yu M, Wu X, Peng L, Yang M, Zhou H, Xu J, et al. Inhibition of Bruton’s Tyrosine kinase alleviates monocrotaline-induced pulmonary arterial hypertension by modulating macrophage polarization. Oxid Med Cell Longev. 2022;2022:6526036. 36071873 10.1155/2022/6526036 PMC9444460

[28] Misharin AV, Morales-Nebreda L, Mutlu GM, Budinger GR, Perlman H. Flow cytometric analysis of macrophages and dendritic cell subsets in the mouse lung. Am J Respir Cell Mol Biol. 2013;49:503–10. 23672262 10.1165/rcmb.2013-0086MA PMC3824047

[29] Hoeve MA, Nash AA, Jackson D, Randall RE, Dransfield I. Influenza virus A infection of human monocyte and macrophage subpopulations reveals increased susceptibility associated with cell differentiation. PLoS One. 2012;7:e29443. 22238612 10.1371/journal.pone.0029443 PMC3251590

[30] Hawse WF, Boggess WC, Morel PA. TCR signal strength regulates akt substrate specificity to induce alternate murine Th and T regulatory cell differentiation programs. J Immunol. 2017;199:589–97. 28600288 10.4049/jimmunol.1700369 PMC5575766

[31] Poltorak M, Meinert I, Stone JC, Schraven B, Simeoni L. Sos1 regulates sustained TCR-mediated Erk activation. Eur J Immunol. 2014;44:1535–40. 24497027 10.1002/eji.201344046

[32] Ivetac I, Gurung R, Hakim S, Horan KA, Sheffield DA, Binge LC, et al. Regulation of PI(3)K/Akt signalling and cellular transformation by inositol polyphosphate 4-phosphatase-1. EMBO Rep. 2009;10:487–93. 19325558 10.1038/embor.2009.28 PMC2680870

[33] Barrio E, Vecino R, Sanchez-Moran I, Rodriguez C, Suarez-Pindado A, Bolanos JP, et al. Preconditioning-activated AKT controls neuronal tolerance to ischemia through the MDM2-p53 Pathway. Int J Mol Sci. 2021;22:7275. 34298892 10.3390/ijms22147275 PMC8304232

[34] Maddox L, Schwartz DA. The pathophysiology of asthma. Annu Rev Med. 2002;53:477–98. 11818486 10.1146/annurev.med.53.082901.103921

[35] Galli SJ, Tsai M, Piliponsky AM. The development of allergic inflammation. Nature. 2008;454:445–54. 18650915 10.1038/nature07204 PMC3573758

[36] Newcomb DC, Boswell MG, Sherrill TP, Polosukhin VV, Boyd KL, Goleniewska K, et al. IL-17A induces signal transducers and activators of transcription-6-independent airway mucous cell metaplasia. Am J Respir Cell Mol Biol. 2013;48:711–6. 23392574 10.1165/rcmb.2013-0017OC PMC3727878

[37] Molet S, Hamid Q, Davoine F, Nutku E, Taha R, Page N, et al. IL-17 is increased in asthmatic airways and induces human bronchial fibroblasts to produce cytokines. J Allergy Clin Immunol. 2001;108:430–8. 11544464 10.1067/mai.2001.117929

[38] Capitini CM, Chisti AA, Mackall CL. Modulating T-cell homeostasis with IL-7: preclinical and clinical studies. J Intern Med. 2009;266:141–53. 19623690 10.1111/j.1365-2796.2009.02085.x PMC2797310

[39] He C, Zhou Y, Li Z, Farooq MA, Ajmal I, Zhang H, et al. Co-Expression of IL-7 Improves NKG2D-based CAR T cell therapy on prostate cancer by enhancing the expansion and inhibiting the apoptosis and exhaustion. Cancers. 2020;12:1969. 32698361 10.3390/cancers12071969 PMC7409228

[40] Amos CL, Woetmann A, Nielsen M, Geisler C, Odum N, Brown BL, et al. The role of caspase 3 and BclxL in the action of interleukin 7 (IL-7): a survival factor in activated human T cells. Cytokine. 1998;10:662–8. 9770327 10.1006/cyto.1998.0351

[41] Nagata S. Apoptosis and clearance of apoptotic cells. Annu Rev Immunol. 2018;36:489–517. 29400998 10.1146/annurev-immunol-042617-053010

[42] Ly JD, Grubb DR, Lawen A. The mitochondrial membrane potential (deltapsi(m)) in apoptosis; an update. Apoptosis. 2003;8:115–28. 12766472 10.1023/a:1022945107762

[43] Zhang S, Fan S, Wang Z, Hou W, Liu T, Yoshida S, et al. Capecitabine regulates HSP90AB1 Expression and induces Apoptosis via Akt/SMARCC1/AP-1/ROS Axis in T cells. Oxid Med Cell Longev. 2022;2022:1012509. 35368874 10.1155/2022/1012509 PMC8970866

[44] Shang J, Gao ZY, Zhang LY, Wang CY. Over-expression of JAZF1 promotes cardiac microvascular endothelial cell proliferation and angiogenesis via activation of the Akt signaling pathway in rats with myocardial ischemia-reperfusion. Cell Cycle. 2019;18:1619–34. 31177938 10.1080/15384101.2019.1629774 PMC6619954

[45] Zeng C, Zhang L, Luo C, Yang C, Huang X, Fan L, et al. A stratification model of hepatocellular carcinoma based on expression profiles of cells in the tumor microenvironment. BMC Cancer. 2022;22:613. 35659630 10.1186/s12885-022-09647-5 PMC9167552

[46] Fan JW, Fan Y, Wan ZL, Yan L, Li YM, Bai YJ, et al. The ratio of CD226 and TIGIT Expression in Tfh and PD-1(+)ICOS(+)Tfh cells are potential biomarkers for chronic antibody-mediated rejection in kidney transplantation. J Immunol Res. 2022;2022:5326083. 35733922 10.1155/2022/5326083 PMC9206998

[47] Conner M, Hance KW, Yadavilli S, Smothers J, Waight JD. Emergence of the CD226 axis in cancer immunotherapy. Front Immunol. 2022;13:914406. 35812451 10.3389/fimmu.2022.914406 PMC9263721

[48] Xie Y, Zhang Y, Zhu T, Ma J, Duan C, Yang L, et al. CD226 deficiency alleviates murine allergic rhinitis by suppressing Group 2 innate lymphoid cell responses. Mediators Inflamm. 2022;2022:1756395. 35846105 10.1155/2022/1756395 PMC9283078

[49] Bachelet I, Munitz A, Mankutad D, Levi-Schaffer F. Mast cell costimulation by CD226/CD112 (DNAM-1/Nectin-2): a novel interface in the allergic process. J Biol Chem. 2006;281:27190–6. 16831868 10.1074/jbc.M602359200

