# Supplementary material for: CD226 implicated in Akt-dependent apoptosis of CD4+ T cell contributes to asthmatic pathogenesis
Source: Cell Death Dis. 2024 Sep 30;15(9):705. doi: 10.1038/s41419-024-07080-z (PMC11442704; doi:10.1038/s41419-024-07080-z)
Supplement: Supplementary file 1 — Supplementary Information [file 41419_2024_7080_MOESM1_ESM.docx]

**Supplementary Information**

**Supplementary Figure 1**

**
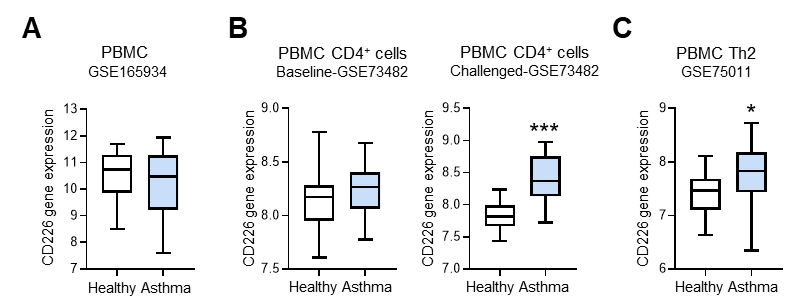
**

**Suppl. Fig. 1. CD226 is upregulated in CD4^+^ T cells from patients with asthma based on bioinformatics analysis.** (A) GSE165934 database analysis for CD226 mRNA expression of PBMCs from patients with asthma and healthy subjects (asthma: n = 10; healthy: n = 9). (B) GSE73482 database analysis for CD226 mRNA expression of peripheral blood CD4^+^ T cells from asthma patients allergic to house dust mite and from healthy subjects (asthma: n = 22; healthy: n = 23); “baseline” refers to the gene expression data obtained immediately after separation of CD4^+^ T cells from patient blood without any additional treatment while “challenged” refers to the gene expression data measured following *in vitro* stimulation with allergen. (C) GSE75011 database analysis for *Cd226* mRNA expression of peripheral blood Th2 cells from asthma patients and healthy subjects (asthma: n = 40; healthy: n = 15). * *P* < 0.05, *** *P* < 0.001 vs. healthy. PBMCs: peripheral blood mononuclear cells; Th: helper T cells.

**Supplementary Figure 2**


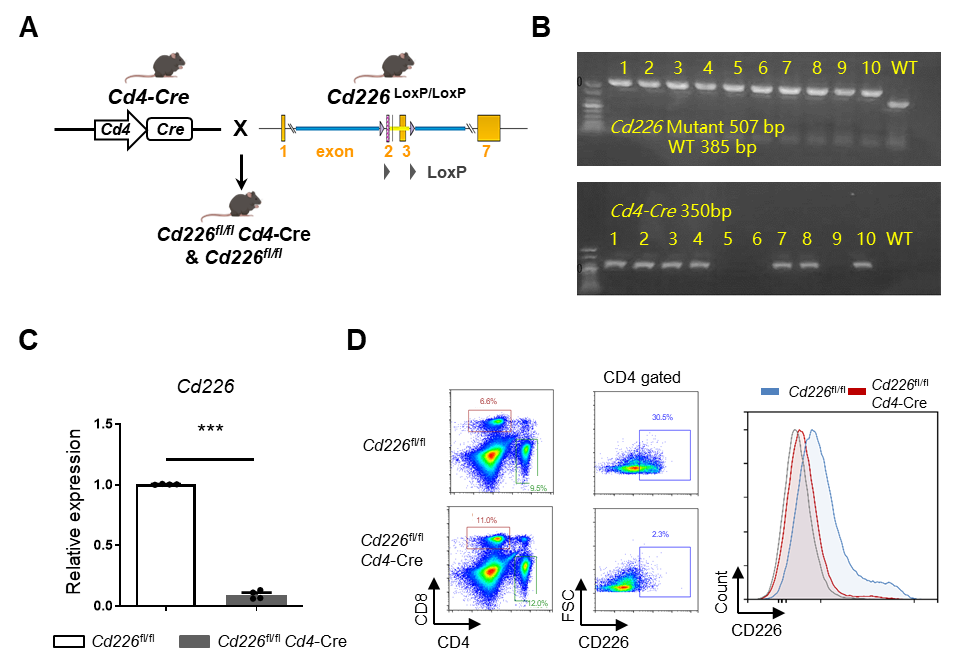


**Suppl. Fig. 2. Generation and identification of conditional CD4^+^ T cell-specific deletion of *Cd226* mice.** (A) Schematic representation of the strategy used to target the exons encoding *Cd226*. (B) Genotyping of *Cd226*^fl/fl^*Cd4*-Cre mice. (C) *Cd226* mRNA levels were assessed via qPCR analysis of CD4^+^ T cells from *Cd226*^fl/fl^*Cd4*-Cre and *Cd226*^fl/fl^ mice. CD4^+^ T cells were isolated from splenocytes using a Dynal mouse T cell negative isolation protocol. Each dot represents an individual mouse. (D) Knockout efficiency of *Cd226* in CD4^+^ T cells was determined using FCM analysis. Data are shown as the mean ± SEM, n = 3 ~ 4 per group. *** *P* < 0.001.

**Supplementary Figure 3**

**
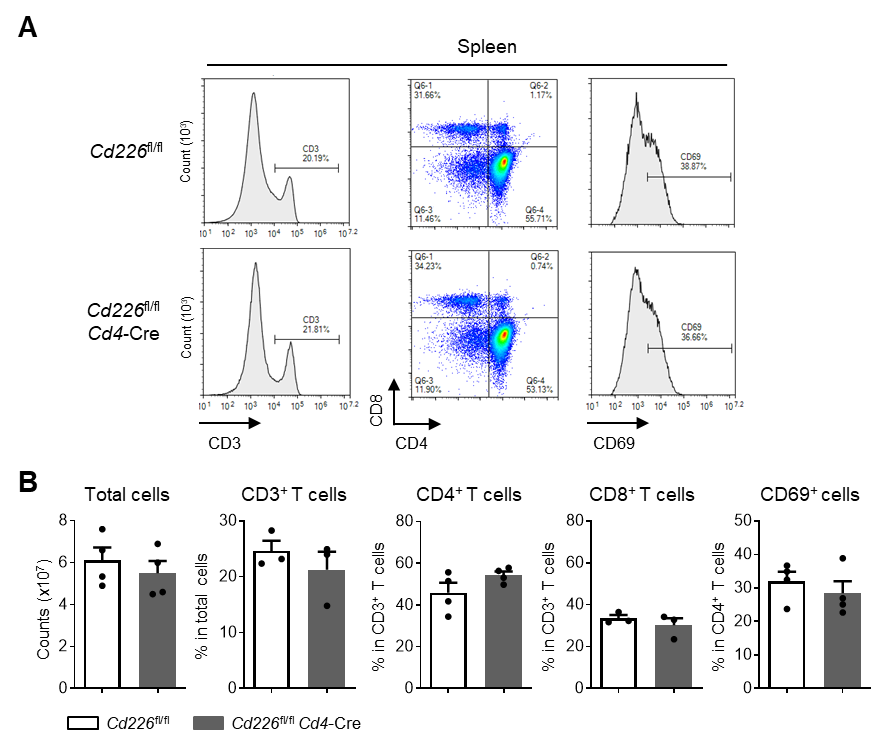
**

**Suppl. Fig. 3. CD226 deficiency in T cells did not affect T cell development in normal mice.** (A) FCM analysis and quantification for evaluating total CD45^+^ leukocyte number, CD3-, CD4-, or CD8-, and CD69-positive populations in the spleen out of *Cd226*^fl/fl^*Cd4*-Cre or littermate *Cd226*^fl/fl^ mice. All displayed FCM plots are CD45^+^ gated. CD4 and CD8 expression is shown as a percentage gated of CD45^+^CD4^+^ splenocytes. CD69 expression is shown as a percentage gated of CD45^+^CD3^+^CD4^+^ splenocytes. Total cells quantification was determined by CountBright Absolute Counting Beads. (B) Quantitative data of FCM analysis. All data are mean ± SEM, each dot represents an individual mouse.

**Supplementary Figure 4**

**
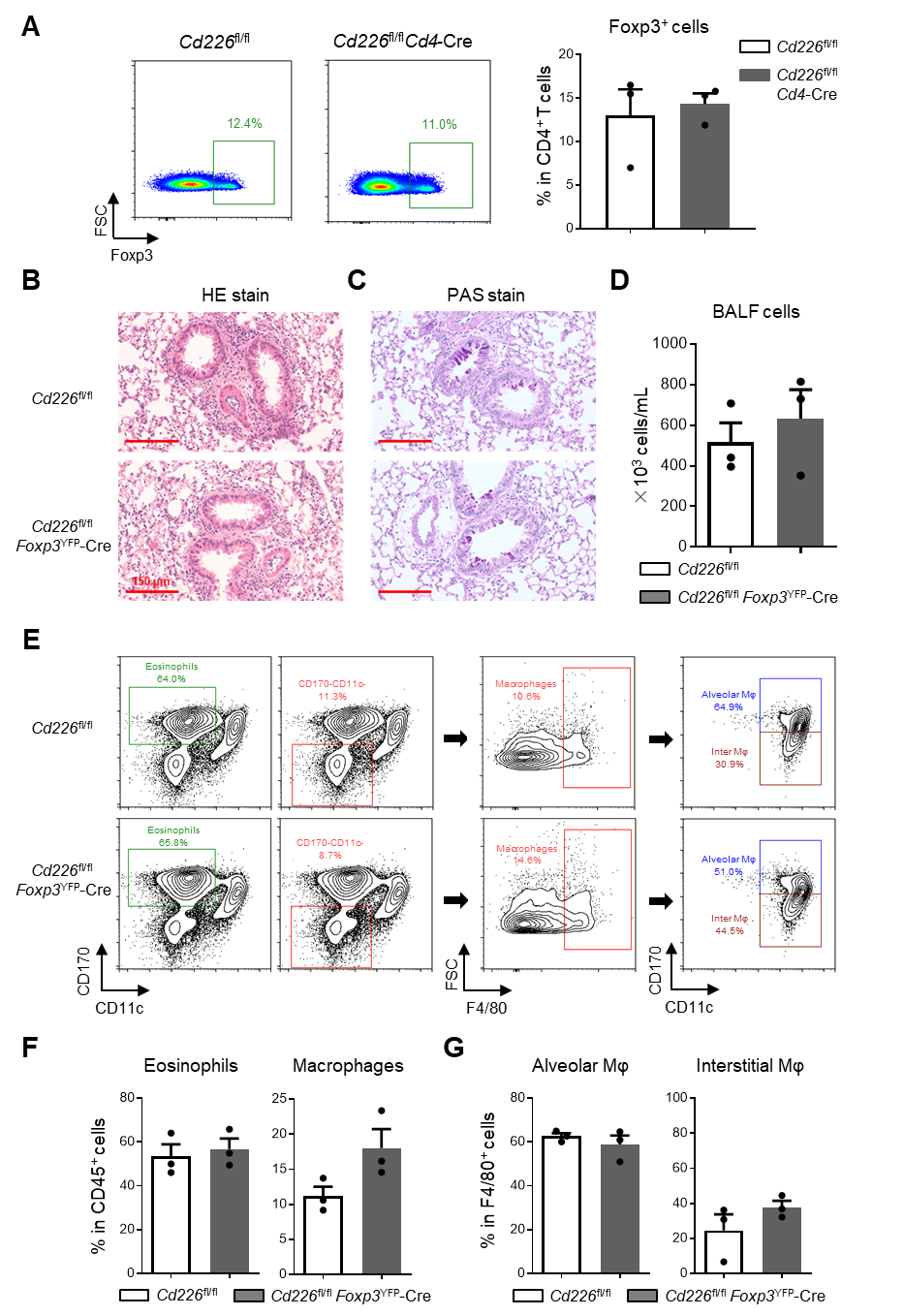
**

**Suppl. Fig. 4. Lack of CD226 on Treg cells did not affect OVA-induced asthmatic mice.** (A) FCM analysis of Foxp3^+^ Treg cells in the spleen of OVA‐challenged *Cd226*^fl/fl^*Cd4*-Cre and *Cd226*^fl/fl^ mice. (B) H&E staining of lungs from *Cd226*^fl/fl^*Foxp3*^YFP^-Cre and littermate mice (*Cd226*^fl/fl^). (C) Lung tissue sections were PAS stained; scale bar = 150 μm. (D) Total cell number in BALF was counted in *Cd226*^fl/fl^*Foxp3*^YFP^-Cre and *Cd226*^fl/fl^ mice. (E) Representative FCM histograms depicting the frequency of eosinophils (CD11c^−^CD170^+^) and macrophages (CD11c^−^CD170^–^F4/80^+^) in BALF; macrophages are further divided into exudate alveolar macrophages (F4/80^+^CD11c^+^CD170^+^) and interstitial macrophages (F4/80^+^CD11c^+^CD170^–^). Displayed FCM plots are CD45^+^ gated. (F) Quantitative data of FCM analysis for eosinophils and macrophages. (G) Quantitative data of FCM analysis for exudate alveolar and interstitial macrophages in BALF. All data are mean ± SEM, each dot represents an individual mouse. Treg: regulatory T cells; OVA: ovalbumin; H&E: hematoxylin and eosin; PAS: periodic acid–Schiff; BALF: bronchoalveolar lavage fluid; FCM: flow cytometry; Mφ: macrophage.

**Supplementary Figure 5**


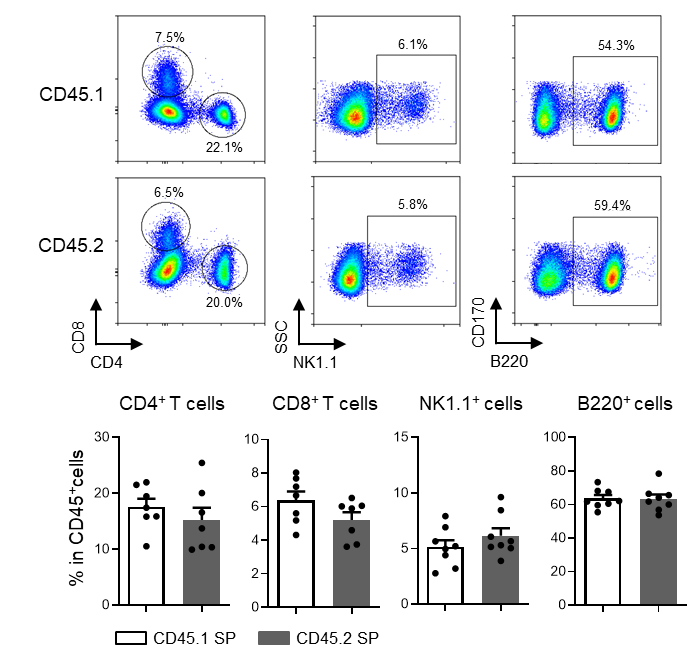


**Suppl. Fig. 5. CD226 deficiency in T cells did not affect T cell development in normal mice.** FCM was performed to assess B, NK, CD4^+^, and CD8^+^ T cells among CD45.1 (WT) or CD45.2 (KO) splenocytes from bone marrow chimera mice. FCM plots are CD45^+^ gated. All data are mean ± SEM, each dot represents an individual mouse. FCM: flow cytometry.

**Supplementary Table 1. qPCR primer sequences**

| **Gene** | **Forward (5ʹ–3ʹ)** | **Reverse (5ʹ–3ʹ)** |
| --- | --- | --- |
| *Il2* | GCGGCATGTTCTGGATTTGACTC | CCACCACAGTTGCTGACTCATC |
| *Il4* | ATCATCGGCATTTTGAACGAGGTC | ACCTTGGAAGCCCTACAGACGA |
| *Il5* | GATGAGGCTTCCTGTCCCTACT | TGACAGGTTTTGGAATAGCATTTCC |
| *Il13* | AACGGCAGCATGGTATGGAGTG | TGGGTCCTGTAGATGGCATTGC |
| *Il17a* | CAGACTACCTCAACCGTTCCAC | TCCAGCTTTCCCTCCGCATTGA |
| *Ifng* | CAGCAACAGCAAGGCGAAAAAGG | TTTCCGCTTCCTGAGGCTGGAT |
| *Cd226* | CAGACATTGGCATCTACTCCTGC | GACATCTTGTCCAGGTTCTGCAG |
| *β-actin* | CATTGCTGACAGGATGCAGAAGG | TGCTGGAAGGTGGACAGTGAGG |
