# Supplementary figures and images for: CD226 implicated in Akt-dependent apoptosis of CD4+ T cell contributes to asthmatic pathogenesis
Source: Cell Death Dis. 2024 Sep 30;15(9):705. doi: 10.1038/s41419-024-07080-z (PMC11442704; doi:10.1038/s41419-024-07080-z)

Figure 6D

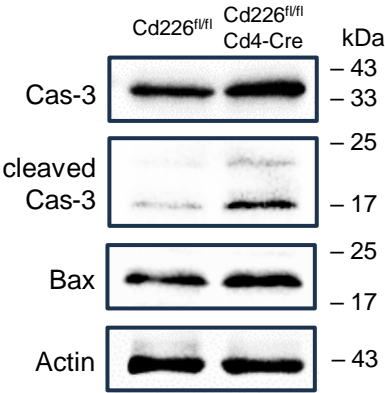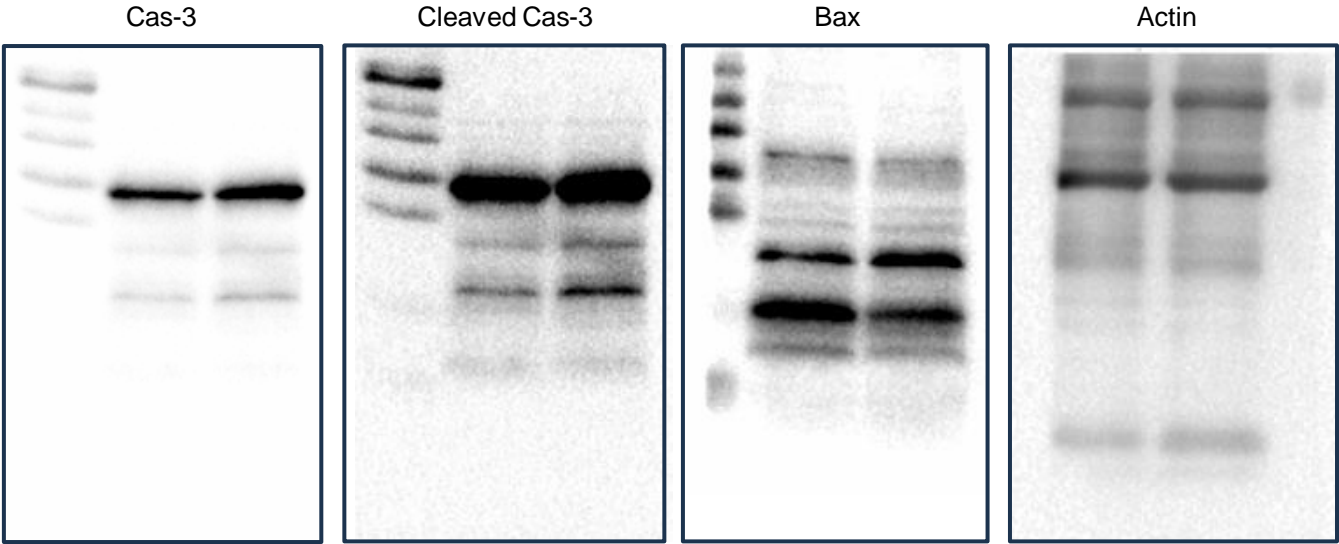

Figure 7A

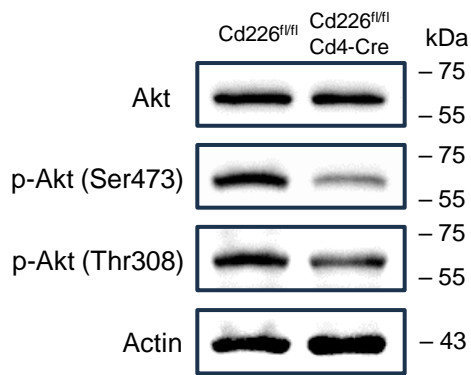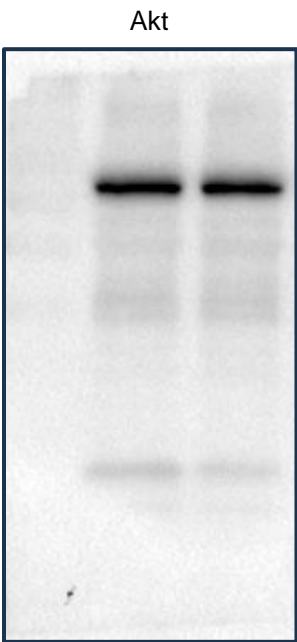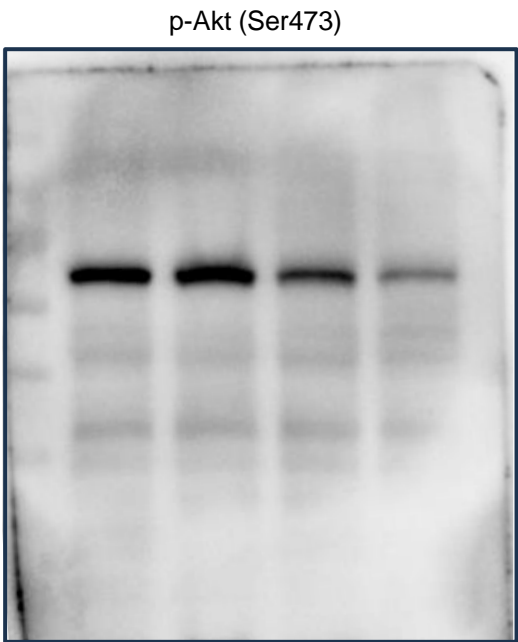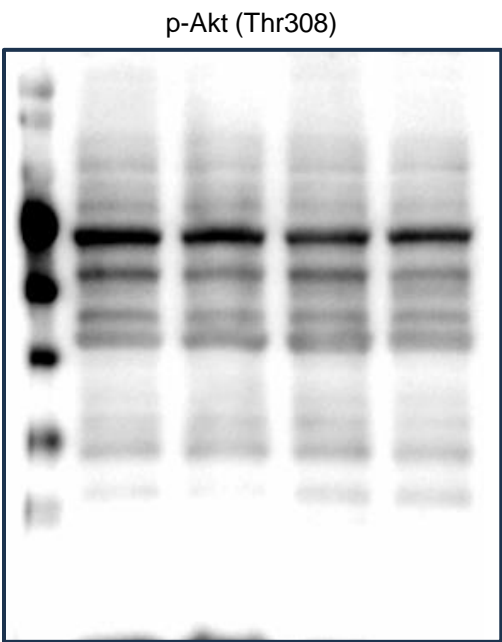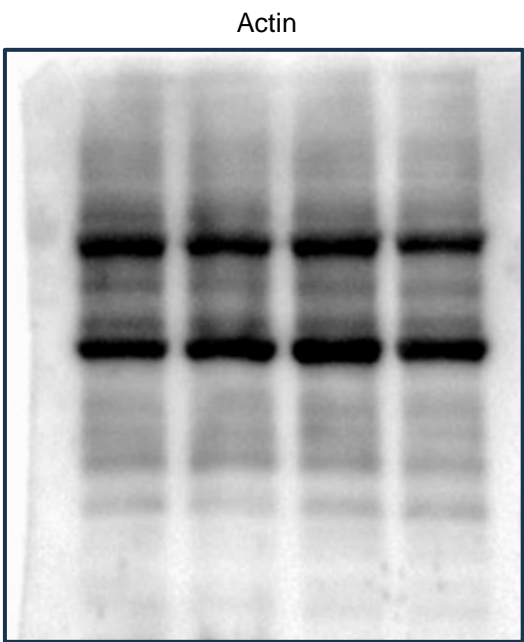

Supplement: Supplementary file 2 — Supplementary Information-WB original images [file 41419_2024_7080_MOESM2_ESM.pdf]
